# Supplementary material for: Towards unified AI-driven fracture mechanics: the extended deep energy method (XDEM)
Source: Nat Commun. 2026 Aug 15;17:8492. doi: 10.1038/s41467-026-76748-1 (PMC13484471; doi:10.1038/s41467-026-76748-1)
Supplement: Supplementary file 3 [file 41467_2026_76748_MOESM3_ESM.zip › pic/Results/Stress_intensity_factor/Mixed/beta_45/crack_k_history_beta45.pdf]

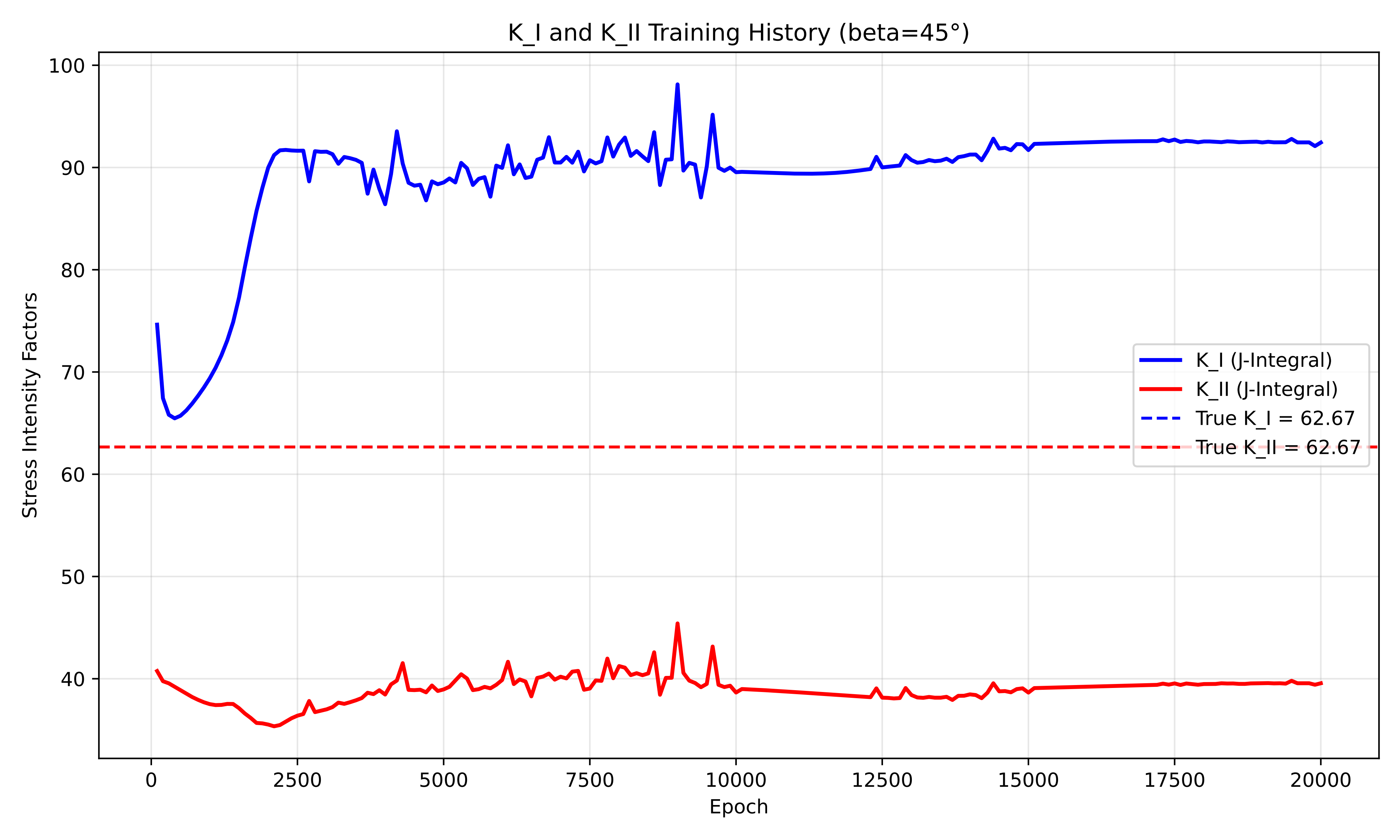
K_I and K_II Training History (beta=45°)
Stress Intensity Factors
Epoch
100
90
80
70
60
50
40
0
2500
5000
7500
10000
12500
15000
17500
20000
K_I (J-Integral)
K_II (J-Integral)
True K_I = 62.67
True K_II = 62.67
